# A hypothesis of couplet molecules and couplet cells in gastric function and an association with *Helicobacter pylori*

**DOI:** 10.1186/s12876-016-0429-0

**Published:** 2016-02-16

**Authors:** Cyril John Craven

**Affiliations:** Queensland University of Technology, Brisbane, Australia

**Keywords:** G-cells, Enterochromaffin-like cells, Gastrin, Histamine, Couplet molecules and couplet cells

## Abstract

**Background:**

Gastrin, from G-cells, and histamine, from enterochromaffin-like (ECL) cells, are two of the hormones that regulate gastric activity.

**Discussion:**

It is proposed that the G-cells and the ECL cells are coupled by the couplet molecules gastrin and histamine and by a prior asymmetrical cell division. The gastrin (from G-cells) stimulates the ECL cells to produce and secrete histamine while, in a reciprocal way, this histamine (from ECL cells), stimulates the G-cells to produce and secrete gastrin. These molecules would also stimulate cell division – the gastrin would stimulate cell division of ECL cells while histamine would stimulate that of G-cells. A chemical complex of gastrin and histamine is postulated as is also the asymmetric cell divisions of precursor cells to produce the coupled G-cells and ECL cells.

**Conclusion:**

There is sufficient evidence to support the feasibility of the model in general, but more direct experimental evidence is required to validate the model as applied here to gastric function.

## Background

Gastric activity is regulated by various hormones including gastrin and histamine. The hypothesis offered here will tie together the activities of these two hormones and the specific cells that produce them, namely G-cells and enterochromaffin-like (ECL) cells, respectively.

**Gastrin** is a hormone that is secreted from G-cells which are mainly in the antrum of the stomach and the duodenum, and it stimulates acid secretion by the parietal cells. There are a number of forms of gastrin including ones with 14, 17 or 34 amino acids, perhaps sulphated, amidated or with an additional glycine at the C-terminus. There are a number of potential receptors for gastrin and its related molecules – cholecystokinin (CCK) A, B and C receptors plus other high-affinity receptors. Of these, CCKB binds sulphated gastrin; CCKC is a low-affinity gastrin binding protein; and there are high-affinity receptors selective particularly for amidated gastrin [1]. The major gastrin receptor (CCKB) is a G-protein coupled receptor and is also found in the CNS.

**Histamine** also acts as a hormone and neurotransmitter. It is produced by enterochromaffin-like (ECL) cells and exerts hormonal control of gastric acidity. It is also produced by mast cells and basophils and triggers an inflammatory response to foreign pathogens. There are four specific types of histamine receptors (H1, H2, H3, H4) and they are 7-transmembrane G-protein coupled receptors [2]. The H2 receptor functions to stimulate gastric acid production by parietal cells.

The model proposed here is an example of a more generic model that has already been proposed as a basis towards an understanding of multicellular organisation and cellular interactions within tissue cells [3]. The base of this model is an initial asymmetric cell division of precursor cells to produce two cell types which share inherent specific cellular communications. Symmetric cell divisions of the two types of cells will produce a mixed cluster of cells with an equilibrium of metabolic processes maintained by control of individual cell activity and of cell numbers. The cell communications are reciprocal and one of the couplet molecules, produced by one cell type, stimulates the growth of the other cell type via a cell receptor. The type and number of cell divisions will be controlled by the levels of the individual molecules and by the level of the complex formed by the couplet molecules. This generic model has been described with specific reference to various diseases including that associated with Helicobacter pylori [3].

The model has already been further elaborated on for insulin and glucagon as couplet molecules, derived from the beta- and alpha-cells of the pancreas [4]. The model is here specifically interpreted to the G-cells and the neighbouring ECL cells and their stimulation by histamine and gastrin respectively where these coupled molecules are produced by the couplet cells reciprocally. The model requires that a special molecule (a Trefone) will be both a proliferator and a secretagogue of the same cell type. For example, gastrin may be such a molecule as it is a potent gastrointestinal trophic agent acting as a growth factor to induce cell proliferation and is also a histamine secretory agent [5].

## Discussion

### Evidence required to support the model in gastric function

**Fig. 1:**
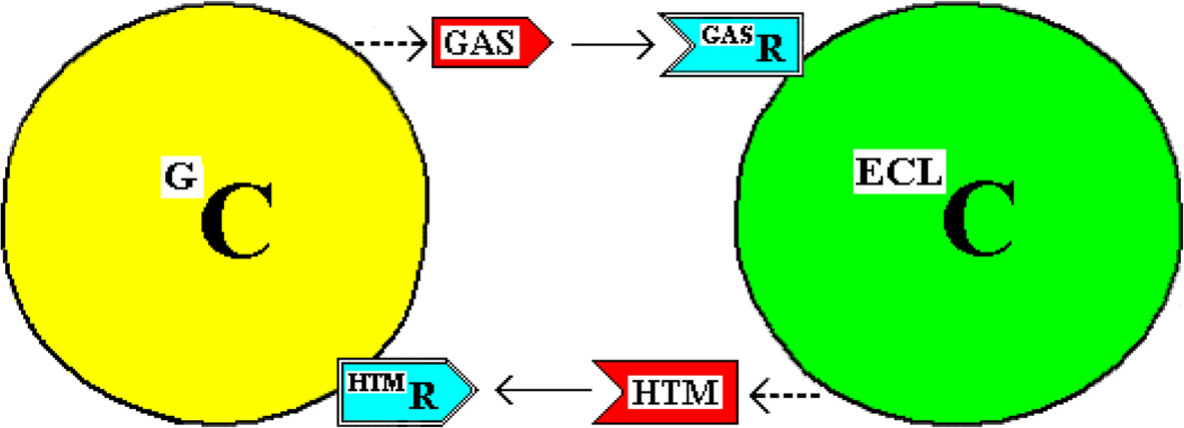
The Simple Interaction of a G-Cell and an ECL Cell. The reciprocal interactions of a G-Cell and an ECL Cell (^G^C and ^ECL^C) are illustrated. The stimulatory effects of Gastrin (GAS) and Histamine (HTM) occur by acting via cell membrane receptors ^GAS^R and ^HTM^R

The model proposed in reference [3] has **C**ouplet molecules (“**T**refones”) produced by couplet **C**ells and is referred to as the CTC model. The couplet cells produced by the asymmetric cell division of a precursor cell are referred to as the a-Cell and the i-Cell. Gastrin and histamine would be Trefone couplets - two interacting, cell-stimulating molecules produced by couplet cells that form a complex described in the generic model as a “Trefone Couplet Complex” This couplet complex (TCC), together with the individual molecules, control cell division*.* The G-Cell and the ECL cell are the cell couplets and, arbitrarily in this proposal, the G-cell is the a-Cell which produces gastrin (^a^T) and the ECL cell is the i-Cell which produces histamine (^i^T). Reciprocal receptors are indicated in Fig. 1.

Thus for the proposed couplet cells (G-cells and ECL cells) with a molecular couplet of gastrin and histamine, the following would be expected:-

  Gastrin binds histamine to form a complex.
  (i).     G-cells have receptors for histamine.
  (ii).     Histamine normally stimulates proliferation of G-cells.
  (iii).     Histamine inhibits proliferation of G-cells when both histamine and gastrin are high.
  (iv).     Histamine stimulates production/secretion of gastrin by G-cells.
  (i).     ECL cells have receptors for gastrin.
  (ii).     Gastrin normally stimulates proliferation of ECL cells.
  (iii).     Gastrin inhibits proliferation of ECL cells when both gastrin and histamine are high.
  (iv).     Gastrin stimulates production/secretion of histamine by ECL cells.
  G-cells and ECL cells each have a receptor for the gastrin: histamine complex.

Evidence to support this model is offered.

***The evidence:-***

***(1) Gastrin (GAS) binds Histamine (HTM)***

There is no evidence for this, known to be recorded. Were studies to be conducted to assess this potential binding, all of the five active forms of gastrin would need to be considered to discover the actual couplet of histamine (ie. the Trefone). Progastrin, the C-terminal Gly-extended gastrins (G34-Gly and G17-Gly) and the C-terminal amidated gastrins (G34 and G17), all have some biological activity [6].

***(2) (i) G-Cells have receptors for Histamine***

Rabbit G-cells have HTM H2-receptors [7] and the cells release gastrin upon HTM stimulation. This receptor would function in a different way in G-cells compared to H2-receptors in parietal cells where the receptor, with HTM bound, stimulates acid production.

***(ii) Histamine stimulates proliferation of G-Cells***

HTM stimulates proliferation of a human gastric adenocarcinoma subline (MKN45G) which itself produces GAS and which therefore could be a model for G-cells [8]. HTM also increased the proliferation of grafted MKN45G tumour tissue in nude mice [8]. HTM it is also known to be a proliferative stimulant for some other cells (eg. airway smooth muscle cells [9], cultured rat thymic epithelium cells [10] and a Leydig cancer cell line [11]).

***(iii) Histamine inhibits proliferation of G-Cells***

HTM is not known to be an inhibitor of G-cells but it is an inhibitor for other cells (eg. colorectal [12], pancreatic carcinoma cells [13]). For the latter, HTM concentrations higher than 1 μmol · L^−1^ inhibited clonogenic growth but nanomolar HTM doses stimulated cell proliferation [14] Within the CTC model, HTM would inhibit proliferation of G-cells when both HTM and gastrin are high but such experiments have not been reported for G-cells.

***(iv) Histamine stimulates production of Gastrin by G-Cells***

HTM stimulates release of GAS from G-cells via H2-receptors, as was previously noted [7].

***(3) (i) ECL cells have receptors for Gastrin***

ECL cells have receptors for GAS [15], and the mRNA for the cholecystokinin B/gastrin receptor is present in ECL cells of the human stomach [16].

***(ii) Gastrin stimulates proliferation of ECL Cells***

GAS stimulates the proliferation rate of both ECL cells and of stem cells in the oxyntic mucosal progenitor zone of the rat stomach [17, 18]. GAS also induces ECL cell proliferation in cell culture [19] and it has a specific proliferative effect on rat ECL cells [20]. Amidated gastrin causes increased proliferation of ECL cells in the oxyntic mucosa of the stomach of mice [21]. Antrectomy in rats causes atrophy of the oxyntic gland mucosa [22].

***(iii) Gastrin inhibits proliferation of ECL cells when [GAS] and (HTM] are high***

GAS inhibition of ECL cells is not recorded but GAS does inhibit proliferation of colon cancer cells [23] although the level of HTM is not known here.

***(iv) Gastrin stimulates production of HTM by ECL Cells***

Gastrin stimulates the synthesis of HTM [24, 25] and GAS stimulates the release of histamine from gastric ECL cells in cell culture [19] and from rabbit fundic mucosal cells enriched in ECL cells [26]. (That GAS stimulates acid production by parietal cells is independent of the proposed model.)

***(4) The G-cells and ECL cells would each have a receptor for the gastrin:histamine complex.***

GAS has four cell receptors with variable binding to gastrin variants [1] and one could possibly bind a gastrin:histamine complex (TCC). Similarly, HTM also has four (H1, H2, H3 and H4) receptors [2] and one could possibly bind the TCC.

### Overview

In this model, gastrin and histamine are stimulants (Trefones) of the ECL cells and the G-cells, respectively. Each cell, via the Trefone it produces, has a stimulating effect on the cell activity and on the cell division of the couplet cell. For example, gastrin stimulates the activity of the ECL cells and stimulates the proliferation rate of both ECL cells and stem cells [17]. An activated ECL cell becomes a hypertrophic cell within a week of exposure to high levels of gastrin and the cell division rate is maximal after about 10 days of hypergastrinemia [27]. Indeed, the histamine-releasing and the trophic effects of gastrin may be mediated via the same gastrin receptor [18].

With adequate nutrients, each Trefone will initially stimulate the recipient cells to produce more of the couplet stimulant and grow in size if necessary. Further, each cell, sustained with adequate nutrients and other growth enhancers will gain competence to progress to cell division and to divide symmetrically or asymmetrically, dependent on the level of the Trefones. In the current model, whether a cell divides and the type of division is contingent on the concentration of the gastrin:histamine complex. The calculated data relevant to this contingency is tabulated in Additional File Six of reference [3]. As an example, if the ECL cell detects a low level of free gastrin, then the response depends on the concentration of the complex of gastrin:histamine. A low, medium or high concentration of complex would signal underactive, duly active or overactive status of the ECL cell itself which has produced low, medium and high levels of total histamine respectively. The cell then has a measure of the amount of histamine relative to the amount of gastrin to allow a decision on whether the two hormones (and their source cells) are in harmony or not. If there is a sustained imbalance, a cellular decision, for each cell type, may then be to divide symmetrically, asymmetrically or with dedifferentiation or with transdifferentiation, as appropriate to bring the G-cells and ECL cells back into harmony. Apoptosis is also possible [3]. Gastrin can induce apoptosis in gastric epithelial cells and this contributes to the development of gastric carcinogenesis [28].

**Fig. 2:**
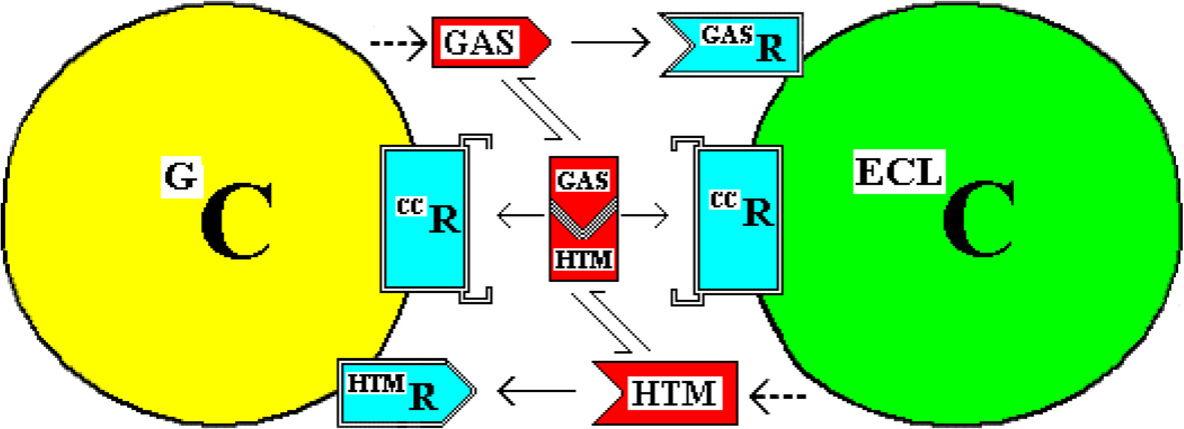
A Possible Complex Interaction of a G-Cell and an ECL Cell. The reciprocal interactions of a G-Cell and an ECL Cell (^G^C and ^ECL^C) are illustrated as in Fig. 1. In addition, this model incorporates a possible extracellular receptor for the Gastrin:Histamine complex (the CC in this particular case)

One possible means by which the cells could measure the gastrin:histamine complex would be to have cell membrane receptors for the complex. This is illustrated in Fig. 2**.** The existence of a membrane receptor is not integral to this hypothesis but there needs to be some intracellular mechanism to measure the level of complex [3].

Once internalised, a component or a signal derived from (i) the gastrin, its receptor and/or the couplet complex in ECL cells or (ii) the histamine, its receptor and/or the couplet complex in G-cells would need to localise to the nucleus to affect gene expression and cell division.

Note that these simple reciprocal interactions are seen to be just a part of a number of potential cellular interactions that produce the complexity of this gastric area.

### The binding of gastrin and histamine to produce a complex – *for investigation*

A metal ion may be involved in the binding of gastrin and histamine.

**Zinc** forms a complex with histamine and zinc-histamine-aspartate and zinc-histamine-glutamate complexes are formed with the respective dicarboxylic acids [29]. Within gastrin (of perhaps 17 amino-acid residues), there are five glutamate residues, in positions 6 to 10, so that a zinc complex with histamine and gastrin is conceivable, given that gastrin binds both divalent and trivalent metal ions [30]. Further, gastrin forms a ternary complex with albumin and various metal ions, and the highest association constant is with zinc [31].

**Iron** could also be involved in a ternary complex. Ferric ions are essential for the biological activity of gastrin (glycine-extended) [32]. Indeed, gastrin binds two ferric ions with high affinity and the glutamate residues of gastrin are involved in the binding of both of these atoms of iron [33]. That it is this ferric complex which is active as a stimulant to proliferation of colonic mucosa [34], may need to be considered. In addition, histamine binds FeIII heme proteins, in particular nitrophorin [35], wherein an aspartate stabilises the complex [36].

### Location of cells and paracrine or endocrine communication

It is generally considered that the ECL cells have no close contact with G-cells. The usual description of the location of these cells is that G-cells are in the antrum and ECL cells are in the corpus/body of the stomach (but accepted to be in the lower third of this area close to the G-cells).

But there is unlikely to be a sharply-defined boundary between the cells in these areas and there is evidence for an overlap or mixed zone of ECL cells and G-cells. G-cells have been mmunocytochemically identified in the antral zone of the rat [37] and gastrin expression has been co-localized with the expression of histidine decarboxylase, an ECL cell marker, in a subset of histaminergic gastric mucosal cells [38].

Gastrin and histamine could interact with the coupling cell via a short diffusion if the cells shared a common location or by more direct cellular contact possibly via cytoplasmic processes [39, 40]. Over larger separatory distances, diffusion directed by microanatomy [41] or either microcirculatory or endocrine transport allow interaction of the molecules with the cells.

### The relationship of this model of gastric function to *Helicobacter pylori*

*H. pylori* could be involved in gastric function in two ways which are compatible with the proposed model. One could be by (a) an oncoprotein virulence factor (CagA), the other by (b) a histamine receptor agonist (methyl histamine).

- One virulence factor of *H. pylori* is the oncoprotein cytotoxin-associated antigen A (CagA). Overexpressed CagA affects various intracellular pathways and is sufficient by itself to induce gastric cancer and other malignancies in transgenic mice [42].
  One mechanism of tumour initiation could involve the specific interaction of CagA with PAR1/MARK kinase [43]. This binding inhibits the kinase activity which is necessary for microtubule stabilisation and consequent epithelial cell polarity [44] and also deregulates SHP-2 phosphatase, an oncoprotein associated with growth regulation and malignancies [45]. Thus CagA affects polarity and subsequent intercellular interactions and alters kinase/phosphatase reactions which could alter cell growth. Gastric carcinogenesis could be due to abnormal proliferation of epithelial cells associated with earlier CagA-induced abnormal intestinal transdifferentiation of cells to produce intestinal metaplasia as an early stage of gastric cancer [45] In addition, PAR1/MARK kinase is one of the six par genes necessary for the asymmetric division of the zygote of *C. Elegans* [46] and these protein kinases are evolutionarily conserved from yeast to humans. If CagA-induced kinase inhibition prevents asymmetric cell division (AsCD) or causes an aberrant AsCD, then the homeostasis of the couplet cells (G-cells and ECL cells) could be disrupted and abnormal proliferation could ensue. In addition, epigenetic alterations (DNA methylations and histone modifications) induced by H. pylori, could contribute to cancer development [47]. However, although this relationship between H. pylori infection and gastric cancer is established, knowledge of the exact mechanism of tumor initiation is lacking [48].
  Within the model proposed here, the mechanism of metaplasia and cancer would be via abnormal AsCD of precursor cells producing abnormal types and/or numbers of G-cells and/or ECL cells. Excess of histamine and/or gastrin or the presence of aberrant/mutated receptor molecules or of molecules which translate the messages of the couplet molecules, would be part of the mechanism.
- In addition to CagA, *H. Pylori* produces N-alpha-methyl histamine (NAMH) [49], which stimulates H3-histamine receptors in gastric mucosa [7, 50] and which stimulates gastrin release from rabbit G-cells via H2- histamine receptors [7].

Within the model proposed here, there are two possibilities:-

(i). If NAMH forms a complex with gastrin, then, with an unregulated supply of NAMH from *H.pylori*, there would be maximal continuous stimulation of gastrin release and of G-cell proliferation. Because of the high level of NAMH, there would be little local free gastrin to stimulate ECL cells. In summary there would be a large numbers of G-cells but few ECL cells. The G-cells may be abnormal if the cell divisions, especially transdifferentiations, are affected by CagA. Gastrin that enters the blood may stimulate hyperacidity of the stomach via the parietal cells.
(ii). If NAMH does not form a complex with gastrin, then there would be a large production of gastrin initially with an associated strong stimulation of histamine release and of ECL cell proliferation. But with a high level of the gastrin:histamine complex, then both cell types would tend to reduce secretion and cell division except that the NAMH would still stimulate the G-cells. Again, CagA may adversely affect cell divisions and transdifferentiation. Histamine, released from mast cells recruited by the *H. pylori* infection [7], may exacerbate this disturbance from normal cell divisions.

## Conclusion

A model is proposed involving asymmetric cell division which produces G-cells and ECL cells which communicate to each other via the secreted couplet molecules of gastrin and histamine. Each will stimulate the cell that it is not secreted from, to stimulate the other cell to secrete more of the couplet molecule and, if this response is inadequate, to stimulate cell division in order to maintain a fixed ratio of gastrin:histamine as assessed by the level of the complex formed by the couplet molecules. Abnormal cell division in Helicobacter infection could be part of the cause of cancer in some cases of this infection.

## Abbreviations

CC: Couplet Complex of Gastrin and Histamine
CTC: Couplet Trefones and Cells
GAS: Gastrin
HTM: Histamine
